# Data-driven wavelet coherence approach to assess neurovascular coupling in neonatal hypoxic–ischemic encephalopathy

**DOI:** 10.1117/1.NPh.13.2.025010

**Published:** 2026-05-28

**Authors:** Srinivas Kota, Soheila Norasteh, Hanli Liu, Esha J. Bhandari, Yu-Lun Liu, Rong Zhang, Lina F. Chalak

**Affiliations:** a University of Texas Southwestern Medical Center, Department of Pediatrics, Division of Neonatal-Perinatal Medicine, Dallas, Texas, United States; b University of Texas Southwestern Medical Center, Peter O’Donnell Jr. Brain Institute, Dallas, Texas, United States; c University of Texas at Arlington, Department of Bioengineering, Arlington, Texas, United States; d University of Texas Southwestern Medical Center, Peter O’Donnell Jr. School of Public Health, Dallas, Texas, United States; e UT Southwestern Medical Center, Department of Neurology, Dallas, Texas, United States

**Keywords:** neonate, hypoxic–ischemic encephalopathy, amplitude-integrated electroencephalogram, cerebral oxygenation, neurovascular coupling, wavelet transform coherence

## Abstract

**Significance:**

Neonatal hypoxic–ischemic encephalopathy (HIE) remains a leading global cause of mortality and morbidity. Neurovascular coupling (NVC), assessed via wavelet transform coherence (WTC) between electroencephalogram and cerebral tissue oxygenation (${\mathrm{SctO}}_{2}$), has shown promise in identifying brain injury severity; however, statistical estimation of WTC was derived using Monte Carlo (MC) simulations with surrogate non-physiological signals.

**Aim:**

To assess NVC using a data-driven method without MC for distinguishing the severity of HIE on the first day of life.

**Approach:**

NVC was assessed on the first day of life with direct data in neonates diagnosed with HIE ranging from mild to severe. Neonates with moderate to severe HIE received therapeutic hypothermia (TH) at 5 h of life (TH group), whereas those with mild HIE did not based on evidence-based protocols (non-TH group). Significant time-scale ranges in WTC were identified using a cluster-based permutation test, and average NVC within these ranges was compared among groups with a linear mixed-effects model over the first 20 h of recording.

**Results:**

A total of 57 full-term neonates with HIE (29 non-TH and 28 TH) were included. The linear mixed-effects revealed a significant interaction between group and time within the 25- to 60-min time (0.28 to 0.67 mHz) scale (p<0.001), indicating reduced NVC with increased encephalopathy severity. Specifically, NVC was significantly reduced in neonates in the TH group (p=0.0103).

**Conclusions:**

We demonstrate that a data-driven approach can significantly distinguish NVC patterns by HIE severity without relying on MC simulations. By enhancing robustness and bedside applicability in the early hours of life, it may support informed decisions regarding initiation of TH to improve outcomes.

## Highlights

• Neurovascular coupling was directly quantified using a wavelet-based approach in neonates with hypoxic–ischemic encephalopathy without Monte Carlo simulations.
• The 25- to 60-min timescale (0.28 to 0.67 mHz) range differentiated TH and non-TH groups.
• Neurovascular coupling (NVC) was significantly lower in the TH group, reflecting greater encephalopathy severity.
• NVC may support early risk stratification and treatment decision-making in HIE.

## 1 Introduction

Hypoxic–ischemic encephalopathy (HIE) affects over a million neonates worldwide.^1^^,^^2^ Although therapeutic hypothermia (TH) treatment significantly improved neurodevelopmental outcomes in moderate-to-severe HIE,^3^^–^^6^ the evolving nature of the injury, makes it extremely difficult to determine the severity of the insult in the critical early hours of life when therapeutic decisions are made.^7^^,^^8^ Neurological examinations in mild HIE may miss subtle abnormalities, especially when asphyxia disrupts neurovascular coupling (NVC).^9^^,^^10^

Our group^11^^–^^15^ and others^16^^–^^20^ have demonstrated that NVC is dynamic physiological biomarker that can assess injury severity in the early hours of life, predict brain abnormalities on magnetic resonance imaging (MRI) at discharge and neurodevelopmental impairment (NDI) at 2 years of age, and characterize physiological responses during neuroprotective therapies.^11^^–^^20^ NVC is assessed using the wavelet transform coherence (WTC) method,^11^^,^^21^ which measures the temporal synchrony between amplitude-integrated electroencephalogram (aEEG) and cerebral tissue oxygenation (${\mathrm{SctO}}_{2}$) measured using near-infrared spectroscopy (NIRS). Monte Carlo simulations are used to assess the statistical significance.^22^ Despite its effectiveness, the Monte Carlo approach carries certain limitations, particularly when adapting for physiological signals. Notably, the simulation does not incorporate real physiological data, which may limit its accuracy and applicability. In addition, the Monte Carlo simulations are not computationally efficient for real-time decision-making.

To support real-time decision-making within this narrow therapeutic window, there is a need for efficient physiological-signal-based methods that can reliably detect clinically meaningful alterations in NVC. A cluster-based permutation approach applied to WTC provides a practical way to identify significant timescale regions in these signals without the computational burden of Monte Carlo simulations. Therefore, the objective of this study was to develop a WTC-based, data-driven approach that does not rely on Monte Carlo simulations to distinguish among HIE groups on the first day of life. The term “data-driven” in this study refers to performing statistical analysis directly on the observed wavelet coherence maps from individual neonates without the use of surrogate data or Monte-Carlo-based significance testing. Statistical inference was conducted using cluster-based permutation testing within a classical hypothesis testing framework.

## 2 Material and Methods

### 2.1 Study Population

Full-term neonates (≥36 weeks of gestational age) with HIE were recruited between 2018 and 2025 at Parkland Hospital, Dallas, Texas, United States, for this prospective cohort study. The study was approved by the Institutional Review Board of the University of Texas Southwestern Medical Center, and written informed consent was obtained from a parent prior to enrollment. Eligible neonates met the following inclusion criteria: (1) history of an acute perinatal event (e.g., cord prolapse, decreased fetal heart rate, or placental abruption), (2) umbilical cord arterial pH or arterial blood gas pH within the first hour of life ≤7.0 or a base deficit ≥15  mmol/L, and (3) clinical signs of encephalopathy. Neonates with genetic or congenital conditions, head circumference<30  cm, or birth weight <1800  g were excluded.

### 2.2 HIE Severity Classification

The severity of HIE was determined by the modified Sarnat examination^23^ performed by trained and certified physicians within 6 h of life. The clinical grade of encephalopathy was assigned based on the maximum number of abnormalities observed and classified as mild, moderate, and severe. Neonates with moderate to severe HIE received TH (TH group) at 33.5°C using Blanketrol (Cincinnati Sub-Zero Products, Cincinnati, Ohio, United States) or Arctic Sun 5000 (Medivance Inc., Louisville, Colorado, United States) as a standard of care for 72 h which was initiated within 6 h of life. Neonates with mild HIE did not receive TH (non-TH group). Neonates initially classified with mild HIE who later developed seizures during the first day of life or showed progression of encephalopathy received late TH^5^ and were classified as the TH group.

#### 2.2.1 Electroencephalogram (EEG) and NIRS data preprocessing

Non-invasive EEG electrodes were placed on the neonates’ scalps using a modified version of the international 10 to 20 system for neonates. For neonates recruited between 2018 and 2019, EEG data were recorded from eight electrodes (Fz, C3, Cz, C4, P3, P4, O1, and O2). For the cohort enrolled between 2023 and 2025, a 19-electrode configuration was used (C3, C4, Cz, F3, F4, F7, F8, Fp1, Fp2, Fz, O1, O2, P3, P4, P7, P8, Pz, T7, and T8). All EEG signals were referenced to the mid-parietal electrode (Pz) during data acquisition. EEG signals were acquired at a sampling rate of 256 Hz using a Nihon Kohden system (Nihon Kohden America, Irvine, California, United States). Simultaneously, ${\mathrm{SctO}}_{2}$ was measured at the center of the forehead using an INVOS™ 4100 to 5100 oximeter (Somanetics, Troy, Michigan, United States) with neonatal sensors, sampled at either 0.21 or 0.03 Hz. Both EEG and ${\mathrm{SctO}}_{2}$ signals were synchronized and recorded using a multi-device integration platform (Moberg Research, Inc., Ambler, Pennsylvania, United States), ensuring precise temporal alignment. Data were stored for offline processing in MATLAB (MathWorks, Inc., Natick, Massachusetts, United States). Duration of multimodal monitoring differed by group (≈24  h in non-TH versus until after rewarming in TH). Analyses were therefore restricted to the first 20 h of recording.

For this study, bipolar EEG signals from the central cross-hemisphere electrodes (C3 to C4) were selected for analysis based on our prior studies.^12^^,^^24^ First, an asymmetric band-pass filter (Parks–McClellan linear phase FIR filter) was applied to attenuate frequencies below 2 Hz and above 15 Hz.^24^^,^^25^ The filtered EEG was then converted to aEEG using procedures adapted from the Washington University-Neonatal EEG Analysis Toolbox.^26^ The aEEG signal was downsampled to match the ${\mathrm{SctO}}_{2}$ sampling rate using a windowed envelope-based technique.^24^ Specifically, a moving time window equivalent to the ${\mathrm{SctO}}_{2}$ sampling interval was used to compute the 90th and 10th percentiles of aEEG values within each window, and their difference was retained as the downsampled value. To remove artifacts, values in the aEEG exceeding 25  μV typically due to motion or electrical interference were identified and replaced using adjacent values. Linear trends were then calculated for both aEEG and ${\mathrm{SctO}}_{2}$ signals. Data points deviating from these trends by more than ±5  μV (aEEG)^26^ or ±20% (${\mathrm{SctO}}_{2}$) ^24^^,^^27^ were also replaced with neighboring values to suppress noise and short-term fluctuations. Signal traces were visually reviewed to confirm quality prior to subsequent analyses. Finally, a second-order polynomial detrending was applied to eliminate residual low-frequency drifts.

### 2.3 Generation of NVC Map Using Wavelet Transform Coherence Without Monte Carlo Simulation

To assess the dynamic interaction between aEEG and ${\mathrm{SctO}}_{2}$, a time-frequency NVC map was generated for each neonate using WTC.^12^^,^^21^ This technique is particularly well-suited for analyzing non-stationary physiological signals such as aEEG and ${\mathrm{SctO}}_{2}$.^11^^,^^12^^,^^24^^,^^28^^,^^29^ WTC analysis was performed using a built-in function *wcoherence* in MATLAB (R2022b; MathWorks, Inc.) to compute the magnitude-squared wavelet coherence ($R^{2}$).^22^^,^^30^ In this framework, the first step is to characterize each signal using the continuous wavelet transform (CWT). The wavelet transform decomposes a time series into a two-dimensional timescale representation. It uses wavelet functions that have zero mean and are localized in both time and frequency. The *wcoherence* function in MATLAB employs the analytic Morlet wavelet as the default mother wavelet because it provides an effective balance between temporal and spectral localization. The analysis used default parameter settings, including the analytic Morlet wavelet, 12 voices per octave to define logarithmic scale resolution, and scale smoothing implemented using a moving average filter with the number of scales equal to the number of voices per octave. The degree of localization is determined by the scale parameter (s), which controls the dilation or compression of the wavelet function. Larger scales correspond to dilated wavelets that emphasize lower-frequency components, whereas smaller scales represent compressed wavelets that capture higher-frequency activity. The transform is computed by convolving the Morlet wavelet, dilated or compressed across a range of scales, with the aEEG signal X(n) and the ${\mathrm{SctO}}_{2}$ signal Y(n). This produces their respective wavelet coefficients $W_{x}(s,n)$ and $W_{y}(s,n)$, where s denotes scale and n denotes time.

The next step is to evaluate the interaction between the two signals by calculating the cross-wavelet transform. This is obtained by multiplying the wavelet coefficients of aEEG $\left(W_{x}(s,n)\right)$ with the complex conjugate of those of ${\mathrm{SctO}}_{2}$ $\left(W_{y}^{*}(s,n)\right)$, yielding $W_{xy}(s,n)=W_{x}(s,n).W_{y}^{*}(s,n)$. The magnitude of this term $\left(|W_{xy}(s,n)|\right)$ reflects the common power exhibited by the two signals across time and scale.

**Fig. 1:**
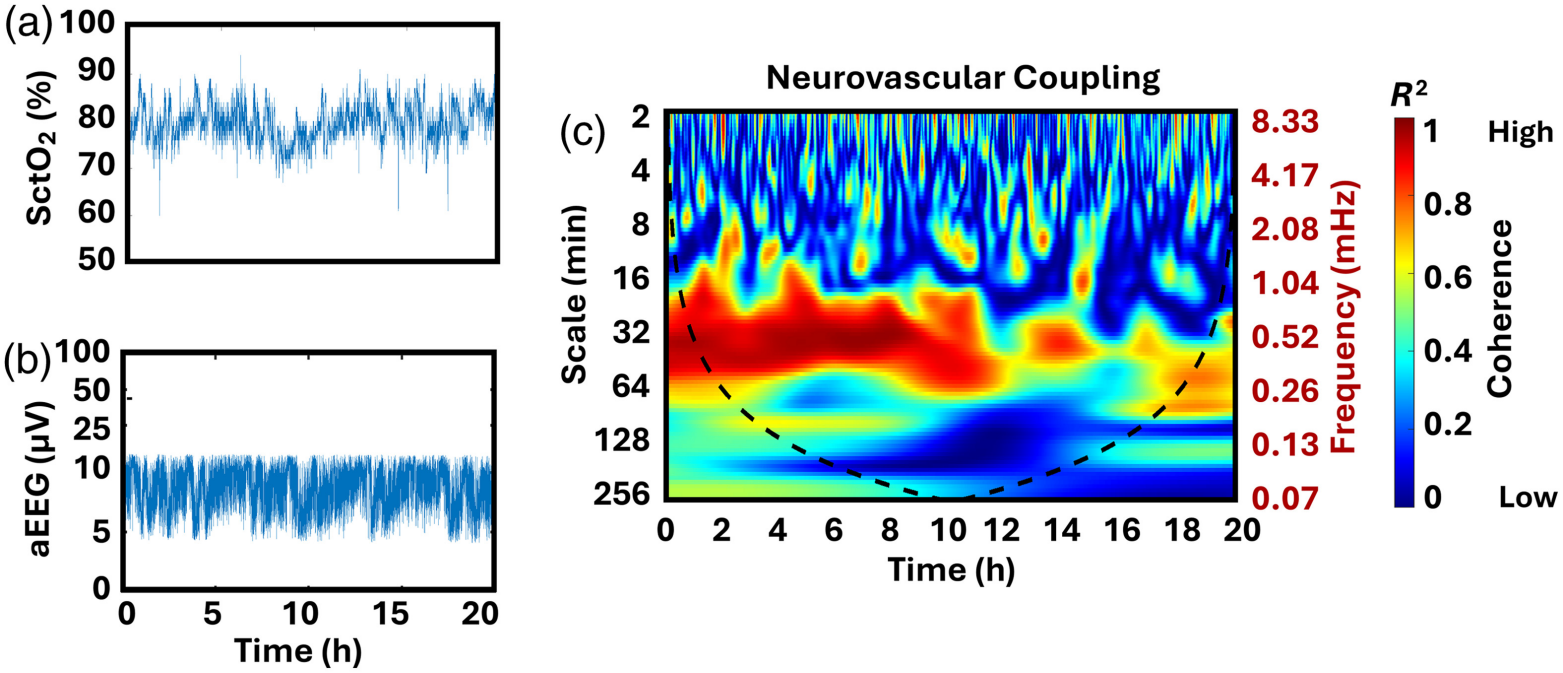
NVC between aEEG and cerebral tissue oxygen saturation (${\mathrm{SctO}}_{2}$). Example of direct data simultaneously recorded ${\mathrm{SctO}}_{2}$ (a) and aEEG (b), with corresponding NVC (c) calculated using WTC for a neonate with mild HIE. The color gradient in the NVC plot represents coherence amplitude: red indicates strong coupling between ${\mathrm{SctO}}_{2}$ and aEEG, and blue indicates the absence of coupling without resorting to Monte Carlo simulation.

Wavelet coherence is then computed by applying smoothing operators to both the cross-wavelet spectrum and the individual wavelet power spectra along the time and scale dimensions. After smoothing, the cross-wavelet energy is normalized by the product of the smoothed wavelet powers. This produces a coherence measure that reflects the strength and consistency of coupling between the two signals across time and frequency. The coherence values range from 0 to 1, with higher values (typically shown as red pixels in the coherence maps) indicating strong coupling, and lower values (blue pixels) reflecting weak or the absence of coupling. These maps provided a detailed visualization of the strength and temporal evolution of NVC across relevant frequency bands for the first 20 h of recording. An illustrative example is provided in Fig. 1, showing the simultaneous recording of ${\mathrm{SctO}}_{2}$ and aEEG in a neonate with mild HIE. The corresponding NVC map derived from these signals is also displayed.

It is important to note that the coherence values presented are raw estimates and were not subjected to statistical testing such as Monte Carlo simulations, surrogate data analysis, or thresholding for significance. Although previous studies by our group^11^^,^^12^^,^^21^^,^^24^^,^^31^ have employed Monte Carlo methods to determine statistical significance, such approaches were not applied in the present analysis.

### 2.4 Statistical Analysis

#### 2.4.1 Maternal and neonatal demographics and clinical characteristics

Maternal and neonatal demographic and clinical characteristics were summarized using descriptive statistics and stratified by treatment group: non-TH (mild HIE) and TH (including mild HIE with progression within the first day of life, moderate, and severe HIE). Continuous variables were expressed as mean ± standard deviation for normally distributed data and as median with first and third quartiles for non-normally distributed data. Categorical variables were reported as counts and percentages. Group comparisons were performed using Student’s t-test or the Wilcoxon rank-sum test for continuous variables, and the $\chi^{2}$ test or Fisher’s exact test for categorical variables, as appropriate.

#### 2.4.2 Cluster-based permutation test

NVC maps were generated for each neonate in the TH and non-TH groups, and group differences were assessed using a cluster-based permutation test in the FieldTrip MATLAB toolbox (version 20250523)^32^ using MATLAB R2023b (MathWorks). Each subject’s NVC matrix (coherence values) was reshaped into FieldTrip-compatible structures with dimensions corresponding to time (0 to 20 h) and scale (1 to 400 min). The analysis was configured to compare the two groups using an independent samples t-test, corrected for multiple comparisons via the cluster-based permutation method with 1000 randomizations.^32^^,^^33^ A cluster-forming threshold of p<0.05 was applied, and statistical significance was evaluated using a one-tailed test. Significant time-scale clusters indicating group-level differences were identified by thresholding the t-statistic map and computing the maximum cluster-level test statistic across permutations. The resulting statistical assessment is marked by black contours on top of the differential WTC map between the two groups, highlighting clusters Z-statistic values. These contours with pixels in red color represent time-scale regions where the TH group exhibited significantly lower NVC compared with the non-TH group. These significant timescale regions were then identified and used for subsequent time-resolved mean NVC analysis.

#### 2.4.3 Time-resolved mean NVC

To further investigate group-level differences in NVC dynamics over time, coherence values were averaged across the identified time-scale range at each time point (0 to 20 h) for both groups of neonates. This produced a time-resolved NVC curve for each subject, representing the mean coherence across the selected scale range over the full 20-h recording period. Group-level NVC trajectories were then obtained by averaging individual curves across subjects in each group. Between-group differences over the full 20-h recording period were assessed using a linear mixed-effects (LME) model.

To further examine the timing of these differences during the early postnatal period, the fitted model was evaluated at predefined 1-h intervals within the 0- to 6-h window using estimated marginal means (*emmeans*). Between-group contrasts were computed at each time point, and a Bonferroni correction was applied to control for type I error arising from multiple testing across seven time points, thereby identifying specific time points at which significant between-group differences were observed. To obtain a single representative measure of overall NVC strength, coherence values were averaged across the significant recording period and restricted to a physiologically relevant scale band. This procedure yielded one mean coherence value per subject. Statistical comparisons between the TH and non-TH groups were conducted using a one-tailed independent two-sample t-test.

All statistical analyses were performed in R (version 4.4.2; R Foundation for Statistical Computing, Vienna, Austria), with marginal means and pairwise contrasts estimated using the “emmeans” package (version 1.11.2-8). A two-sided p value<0.05 was considered statistically significant for all analyses, except for the cluster-based permutation test and the single-measure NVC comparison, for which a one-tailed p value<0.05 was applied.

## 3 Results

Fifty-seven full-term (median gestational age: 39 [38, 40] weeks) neonates were included in this study. Thirty-nine out of 57 of them were identified as mild HIE, 15 were moderate HIE, and 3 were with severe HIE within 6 h of life. TH was initiated at 4±1  h of life for neonates with moderate to severe HIE (TH group). In the mild group, 10 out of 39 neonates progressed between 6 and 24 h of life, and TH was initiated at a median of 11.3 (range: 6.5 to 23.8) h of life and included in the TH group. In total, 28 neonates were included in the TH group and 29 in the non-TH group.

Maternal and neonatal characteristics are presented in Table 1. Maternal ethnicity was predominantly Hispanic (75%). Approximately 65% of neonates were delivered via cesarean section. The most prevalent maternal risk factor in the cohort was pre-eclampsia, reported in 27% of cases overall (18% in the non-TH group and 36% in the TH group; p=0.227), followed by hypertension (21% overall; 18% non-TH, 25% TH; p=0.746) and diabetes (5% overall; 10% non-TH, 0% TH; p=0.237). No statistically significant differences were observed among groups for any maternal risk factor. Regarding labor complications, meconium-stained amniotic fluid and maternal chorioamnionitis were present in 32% of cases. Meconium was noted in 25% of the non-TH group and 39% of the TH group (p=0.391), and chorioamnionitis occurred in 28% and 36%, respectively (p=0.708). Other complications, including placental abruption (9% overall), uterine rupture (5%), and umbilical cord prolapse (4%), showed no significant differences across groups.

**Table 1:** Maternal and neonatal demographics.

Characteristics	Overall cohort	Severity of clinical assessment on day 1		P value
		Non-TH group (mild HIE)	TH group (moderate/severe HIE)	
Total N	57	29	28	
Maternal race/ethnicity: N (%)				0.841
Caucasian non-Hispanic	2 (4)	1 (3)	1 (3)	
Black non-Hispanic	9 (16)	4 (14)	5 (18)	
Hispanic	43 (75)	23 (79)	20 (71)	
Other non-Hispanic	3 (5)	1 (3)	2 (7)	
Delivery mode: N (%)				>0.999
C/S	37 (65)	19 (66)	18 (64)	
Vaginal	20 (35)	10 (35)	10 (36)	
Maternal risk factors: N (%)				
Hypertension	12 (21)	5 (18)	7 (25)	0.746
Diabetes	3 (5)	3 (10)	0 (0)	0.237
Pre-eclampsia	15 (27)	5 (18)	10 (36)	0.227
Labor complications: N (%)				
Meconium	18 (32)	7 (25)	11 (39)	0.391
Umbilical cord prolapse	2 (4)	1 (4)	1 (4)	>0.999
Placental abruption	5 (9)	2 (7)	3 (11)	>0.999
Uterine rupture	3 (5)	1 (4)	2 (7)	>0.999
Maternal chorioamnionitis	18 (32)	8 (28)	10 (36)	0.708
Male: N (%)	35 (61)	19 (66)	16 (57)	0.706
Gestational age (weeks)^b^	39 [38, 40]	39 [38, 40]	39 [38, 40]	0.922
Birth weight (kg)^a^	3.3±0.7 (3.1, 3.5)	3.2±0.5 (3.0, 3.4)	3.4±0.9 (3.0, 3.7)	0.380
Apgar 1 min^b^	3 [1, 4]	3 [3, 5]	1 [1, 3]	<0.001
Apgar 5 min^b^	6 [4, 8]	7 [6, 8]	5 [2, 7]	0.002
Umbilical cord gas pH^a^	7.1±0.2 (7.0, 7.1)	7.1±0.2 (7.1, 7.2)	7.0±0.1 (7.0, 7.1)	0.017
Base deficit^b^	17.1 [14.6, 20.2]	17.1 [13.7, 20.1]	16.8 [14.8, 20.7]	0.622
Disposition				
DOL at discharge^b^	10 [6, 14]	6 [5, 10]	12 [9.8, 18.5]	<0.001
Death prior to discharge, N (%)	2 (3.5)	0 (0)	2 (7.1)	0.237

IQR, interquartile range; SD, standard deviation; CI, confidence interval; DOL, days of life.

P-values were computed using t-tests (normally distributed data) or Wilcoxon rank-sum (skewed data) tests for continuous variables and Fisher’s exact test or chi-square tests for categorical variables.

a Mean ± SD (95% CI) for normally distributed data.

b Median [IQR] for skewed data.

The number of males is slightly higher than that of females in the overall cohort, as well as in both the non-TH and TH groups. No significant differences were observed in birth weight and base deficit across groups. However, Apgar scores at 1 and 5 min and umbilical cord blood pH were significantly lower in the TH group. Length of hospital stay was significantly longer in the TH group. Two neonates with severe HIE died following redirection of care.

**Fig. 2:**
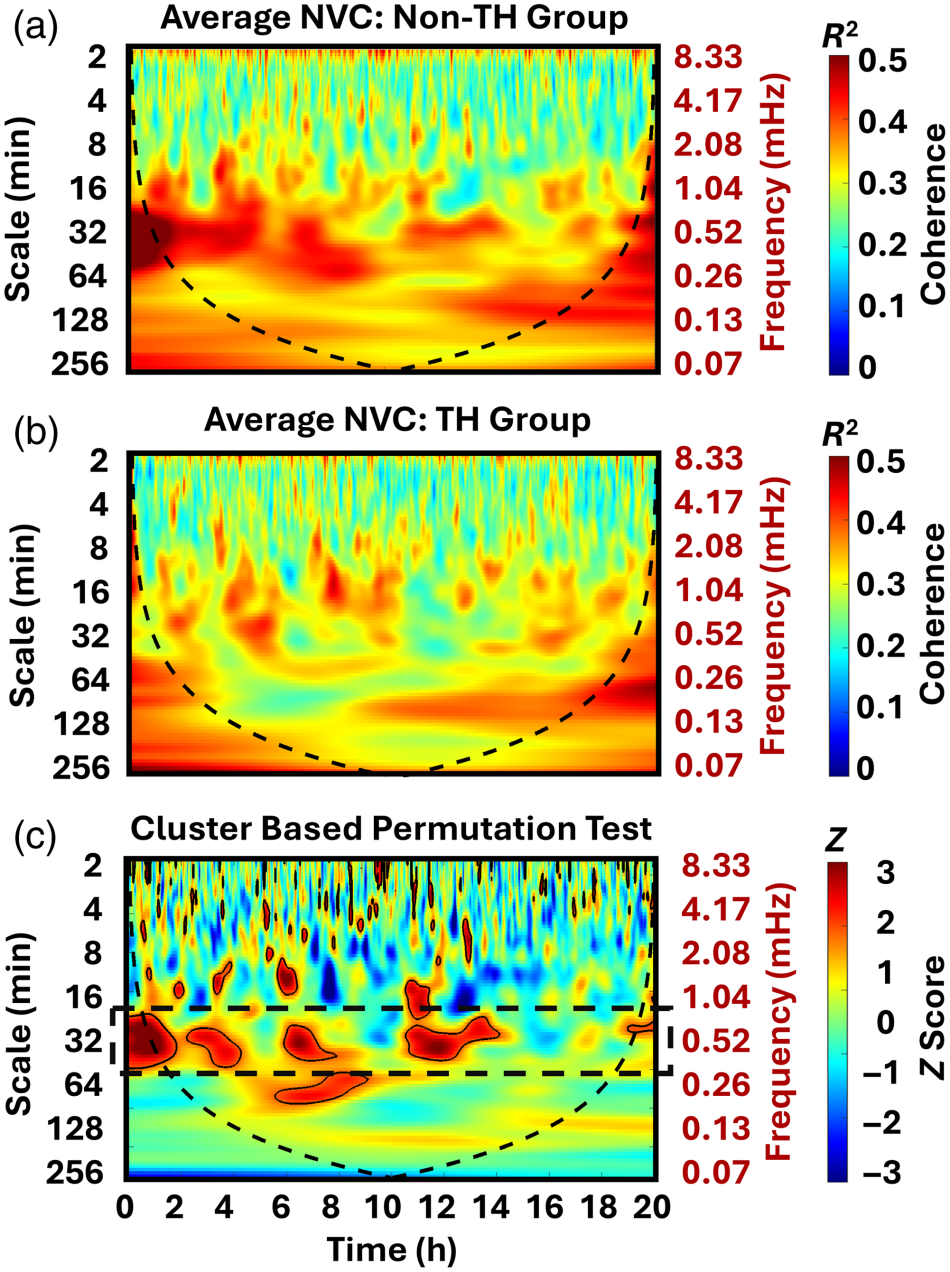
Comparison of NVC in non-therapeutic hypothermia (non-TH) and TH groups. NVC between aEEG and cerebral tissue oxygen saturation (${\mathrm{SctO}}_{2}$), assessed using wavelet transform coherence. The average coherence is shown for neonates in the non-TH (a) and TH (b) groups during the first 20 h of recording. Cluster-based permutation test identified significant timescale regions (c), with Z-statistic values indicating the direction of group differences. Red regions indicate higher NVC in the non-TH group, and blue regions indicate higher NVC in the TH group. A significant difference was observed at timescales of 25 to 60 min (0.28 to 0.67 mHz on Y-axis).

Continuous EEG and NIRS monitoring were initiated at 12±6  h of life. The average NVC maps for the non-TH group are shown in Fig. 2(a), and the average map for the TH group is presented in Fig. 2(b). Additional examples of individual subject NVC maps are provided in Figs. S1 and S2 in the Supplementary Material to illustrate inter-subject variability and the emergence of group-level cluster-based patterns.

**Fig. 3:**
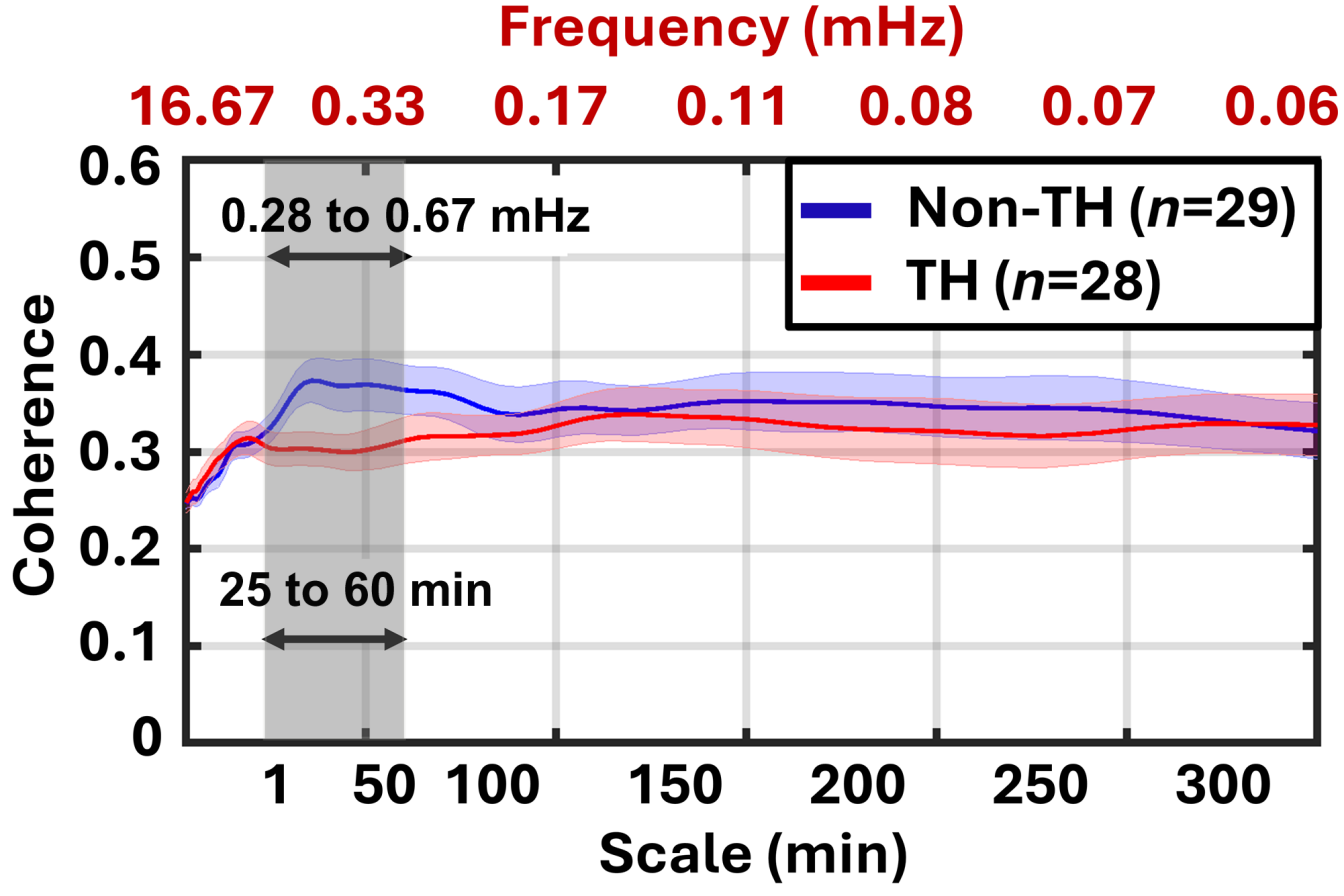
Average coherence values across the 20-h recording period by timescale. The average NVC across all participants is shown, with the non-TH group in blue and the TH group in red. Shaded areas represent the standard error of the mean. Reduced coherence in the TH group is evident at the 25- to 60-min scale (0.28 to 0.67 mHz).

Figure 2(c) displays the Z-statistic values between the two groups after the cluster-based permutation test. Multiple clusters within the 25- to 60-min (0.28 to 0.67 mHz) scale range emerged between 0 and 15 h, indicating that the TH group exhibited significantly lower NVC. To further characterize these differences, Fig. 3 presents the average coherence values across the entire 20-h recording period for each timescale, demonstrating a persistent reduction in NVC strength in the TH group, most notably within the 25- to 60-min time (0.28 to 0.67 mHz) scale range.

**Fig. 4:**
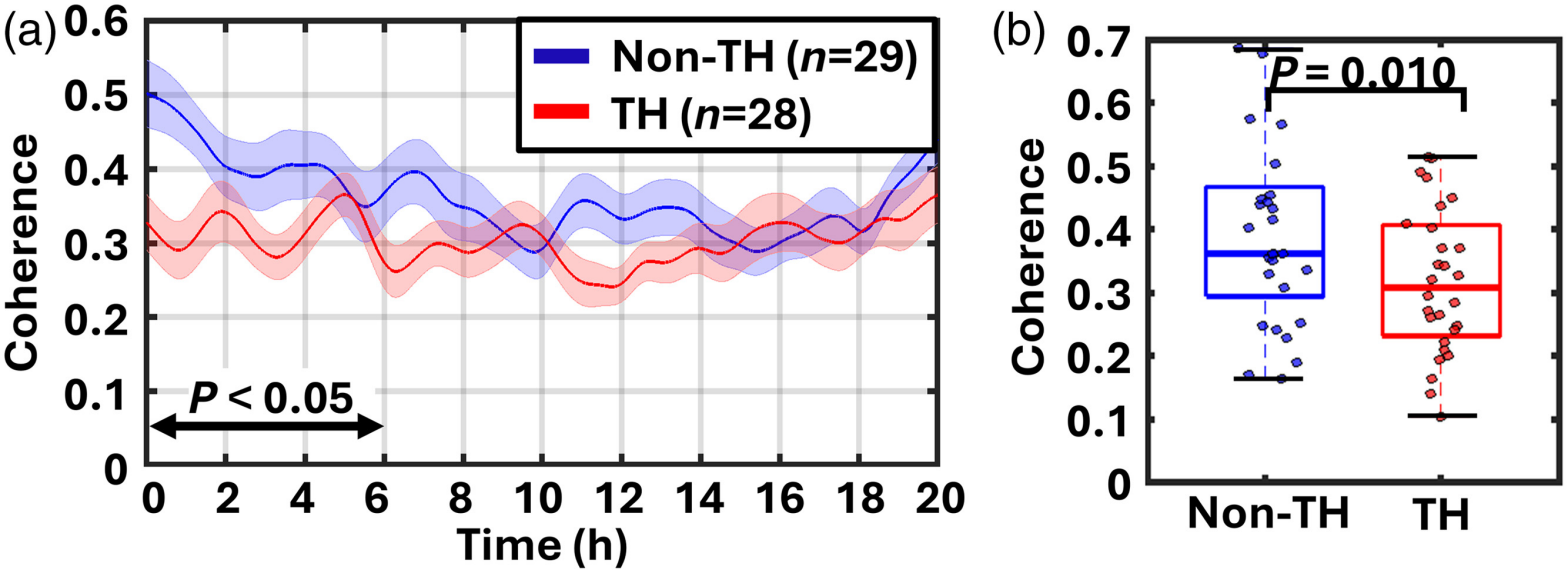
NVC within the 25- to 60-min timescale. (a) Average coherence across participants in the therapeutic hypothermia (TH, red) and non-TH (blue) groups within the 25- to 60-min scale (0.28 to 0.67 mHz) over 20 h. Shaded areas indicate standard error of the mean. Linear mixed-effects modeling showed significant effects of group, time, and group × time interaction (P<0.001). Coherence was lower in the TH group, with group differences during the first 6 h remaining significant after Bonferroni adjustment (p<0.05). (b) Average coherence during 0 to 6 h was significantly lower in the TH group (p=0.010).

For WTC-scaled average over within the 25- to 60-min range, an LME model demonstrated significant main effects of group (Estimate=−0.113, 95%CI:−0.167 to −0.059, p<0.001) and time (Estimate=−0.005, 95%CI:-0.006 to −0.005, p<0.001), as well as a significant group × time interaction (Estimate=0.006, 95% CI: 0.005 to 0.006, p<0.001) over the 20-h period. These results indicate that the TH group exhibited lower NVC values compared with the non-TH group. The divergence in signal trajectories among groups was most significant during the first 6 h of recording. The non-TH group demonstrated significantly higher NVC values than the TH group throughout this period (all Bonferroni-adjusted p<0.05, ranging from <0.001 to 0.031). The estimated group difference declined from 0.113 at baseline to 0.080 at 6 h, as reflected in the group mean curves [Fig. 4(a)]. Figure 4(b) shows the distribution of the average NVC values during the first 0 to 6 h of recording within the 25- to 60-min timescale. As a sensitivity analysis, we repeated the model after excluding the 10 reclassified infants. All effects remained significant and in the same direction, indicating that the findings are robust to reclassification. A one-tailed independent two-sample t-test demonstrated that the mean coherence values were significantly lower in the TH group compared with the non-TH group (p=0.010).

## 4 Discussion and Conclusions

This prospective cohort study introduces a data-driven approach to quantify NVC in neonates with HIE during the first day of life. We demonstrate that NVC was significantly reduced in the TH group compared with the non-TH group, suggesting impaired neurovascular integration in neonates with more severe injury. Clinical decisions regarding TH are currently guided by neurological examination within the first 6 h of life, yet the classification of HIE remains controversial, as injury evolves over time.^7^^,^^33^^–^^35^ Our analysis identified a timescale between 25 and 60 min that reliably distinguished the non-TH and TH groups, with lower coherence in the TH group within the first 6 h of recording. This scale-specific reduction in NVC may reflect disrupted impaired neurovascular dynamics, highlighting the potential utility of NVC metrics as an early, objective biomarker to complement clinical assessment for initiating neuroprotective treatments during early hours of life.

Earlier studies have assessed the significance of time–frequency pixels in NVC maps using surrogate red-noise data, largely due to the limited availability of physiological recordings. For example, Das et al.^12^ reported that an NVC threshold of 10% significance predicted abnormal MRI findings at discharge, and a threshold of 12% predicted NDI at 2 years of age.^13^^,^^14^ In our earlier work, we analyzed 20 h of recording to address the cone of influence; however, such prolonged monitoring may not be feasible for immediate decision-making. To address this, we recently explored a time-resolved zero-padding method applied to shorter data segments and demonstrated that recordings from hours 3 and 4 could effectively stratify the non-TH and TH groups.^27^ Moreover, generating surrogate datasets with 1000 iterations can be computationally demanding, which limits the feasibility of real-time clinical application. This constraint is particularly critical given that decisions regarding TH must be made within the first 6 h of life to optimize outcomes.

Beyond methodological considerations, several studies have investigated the integration of EEG biomarkers with NVC to improve the prediction of HIE severity, MRI abnormalities, and long-term neurodevelopmental outcomes. For example, Hermans et al.^16^ reported that combining NVC with EEG spectral edge frequency (SEF) enhanced the discrimination between mild and moderate–severe HIE on MRI compared with SEF alone. Another study demonstrated that the combination of EEG delta (0.5 to 4 Hz) or total power (0.5 to 20 Hz) with NVC more effectively distinguished neonates who later developed injury.^13^ Furthermore, when clinical assessment within the first 6 h of life was combined with the percentage of significant NVC pixels and EEG delta power, predictive accuracy improved.^14^ Collectively, these studies suggest that continuous multimodal monitoring incorporating both EEG and NVC may provide valuable insights into the true severity of HIE and improve the prediction of both short- and long-term outcomes, thereby supporting timely interventions aimed at optimizing neurodevelopmental prognosis. Unlike earlier methods that relied on Monte Carlo simulations, the present study introduced a data-driven, computationally efficient approach for early risk stratification in HIE, with potential application to real-time assessment of NVC during neuroprotective therapies.^15^

The observed effects at the 25- to 60-min timescale are in agreement with several previous studies, including the initial study by Chalak et al.^11^ using WTC with Monte Carlo simulations demonstrating that NVC in a similar minute scale range stratified neurodevelopmental outcomes at 2 years of age (abnormal in 6 versus normal in 4). A pilot study similarly reported reductions in phase-locking value and mean coherence between the lower envelope of EEG power and ${\mathrm{SctO}}_{2}$ within the same range supporting the role of scale-specific NVC metrics in HIE severity stratification.^36^ Hermans et al.^18^ also used WTC with Monte Carlo simulations in 18 neonates and described a severity-dependent gradient, with strongly positive NVC in mild HIE, lower values in moderate HIE, and severe HIE within the 20- to 80-min range. Consistent with these studies, our observation that reduced coherence in the 25- to 60-min scale-differentiated TH from non-TH groups reinforces the prognostic value of scale-specific NVC as an early biomarker in neonatal encephalopathy. Furthermore, the 25- to 60-min scale range was identified from the full time–frequency map using cluster-based permutation testing rather than being predefined *a priori*. This range corresponds to ∼20 to 48 oscillatory cycles over the 20-h recording duration, thus having effective cycles that warrant the stability and accuracy of coherence estimates.

NVC differed significantly among groups within the first 6 h of recording but with considerable overlap. This overlap may reflect the heterogeneity of HIE and limit discrimination at the individual level. During this early window, when decisions regarding TH are made, NVC may provide supportive information for risk stratification. In this cohort, no newborns were cooled below 32°C, a range associated with suppression of cerebral activity.^37^^–^^39^ Therefore, the reduced NVC in the TH group is more likely to reflect encephalopathy severity rather than an effect of cooling.^40^^–^^43^

In a pilot study, Govindan et al.^17^ quantified NVC over a 40-min dynamic time frame during TH and rewarming, applying a static threshold^44^ to assess significance. They reported that intact survivors showed emergence of NVC, whereas critically ill neonates had no detectable NVC. This important pilot study introduced the concept of threshold-based significance testing. Future studies should focus on defining robust thresholds for coherence significance, which will likely require larger patient cohorts to establish reliability and clinical utility.

Our study has several notable strengths. To our knowledge, this is the first study to compare NVC in TH and non-TH neonates within a relatively large cohort. By focusing on the first day of life, the analysis captures a clinically critical time window when treatment decisions regarding TH must be made. Methodologically, the study introduces a data-driven WTC approach that does not rely on Monte Carlo simulations, thereby reducing computational demands and making real-time bedside implementation feasible. This framework provides an objective, physiology-based complement to early clinical examination and has the potential to scale to multicenter collaborations. Furthermore, it may be extended to the study of other physiological biomarkers, such as cerebral autoregulation,^28^^,^^29^^,^^45^ that can also be evaluated using the WTC framework.

Study limitations include its single-center design, which may limit generalizability, as well as the absence of healthy controls who would not have advanced monitoring data. Although most measured maternal and neonatal characteristics were similar among groups, the modest sample size reduces statistical power to detect subtle imbalances. As an observational cohort without randomization, the study remains susceptible to unmeasured or residual confounding, and the findings should be interpreted descriptively rather than causally. Diagnostic evaluation indicated that the normality assumptions of the linear mixed-effects model were not fully satisfied. Although linear mixed-effects models are generally robust to moderate deviations from normality, these departures may still influence the precision of estimates and the interpretation of p values. We acknowledge that the time-scale band was identified and subsequently analyzed within the same dataset, and therefore, some degree of dependence cannot be fully excluded. Future studies with independent cohorts will be important to confirm the robustness of the identified time-scale band. Expanding the dataset through collaboration with multiple sites in the COOLPRIME trial (NCT04621279) would strengthen our future analyses by incorporating a more diverse population. The cone-of-influence regions were not excluded before performing the cluster-based permutation test. However, the significant cluster within the 25- to 60-min scale range persisted beyond the cone of influence (COI)-affected region, suggesting the findings are unlikely driven by boundary artifacts. The cone-of-influence problem inherent in wavelet analysis remains a methodological constraint, although this can be mitigated using zero-padding.^27^ Evaluating time-resolved zero-padding with shorter data segments, as proposed in this study, will help establish feasibility for early clinical decision-making. Future studies should integrate multimodal biomarkers to create bedside-ready tools for real-time diagnosis and neuroprotective decision-making, with the goal of improving long-term neurodevelopmental outcomes.

This study demonstrates that a data-driven, wavelet-based approach can distinguish NVC patterns in neonates with HIE without relying on Monte Carlo simulations. By enabling scale-specific quantification and improving computational efficiency, the method has the potential for real-time bedside application during the critical early hours of life. These findings highlight NVC as an objective biomarker that may complement clinical examination and inform timely therapeutic decisions.

## Supplementary Material

10.1117/1.NPh.13.2.025010.s01

## Data Availability

Requests for data and computer code access should be directed to the corresponding author and must include a reasonable justification. Access will be granted in accordance with institutional data protection and confidentiality policies.
