# Supplementary material for: Data-driven wavelet coherence approach to assess neurovascular coupling in neonatal hypoxic–ischemic encephalopathy
Source: Neurophotonics. 2026 May 28;13(2):025010. doi: 10.1117/1.NPh.13.2.025010 (PMC13218833; doi:10.1117/1.NPh.13.2.025010)
Supplement: Supplementary file 1 [file NPh_013_025010_SD001.pdf]

A Data-Driven Wavelet Coherence Approach to Assess Neurovascular Coupling in Neonatal Hypoxic-Ischemic Encephalopathy

Supplementary Material

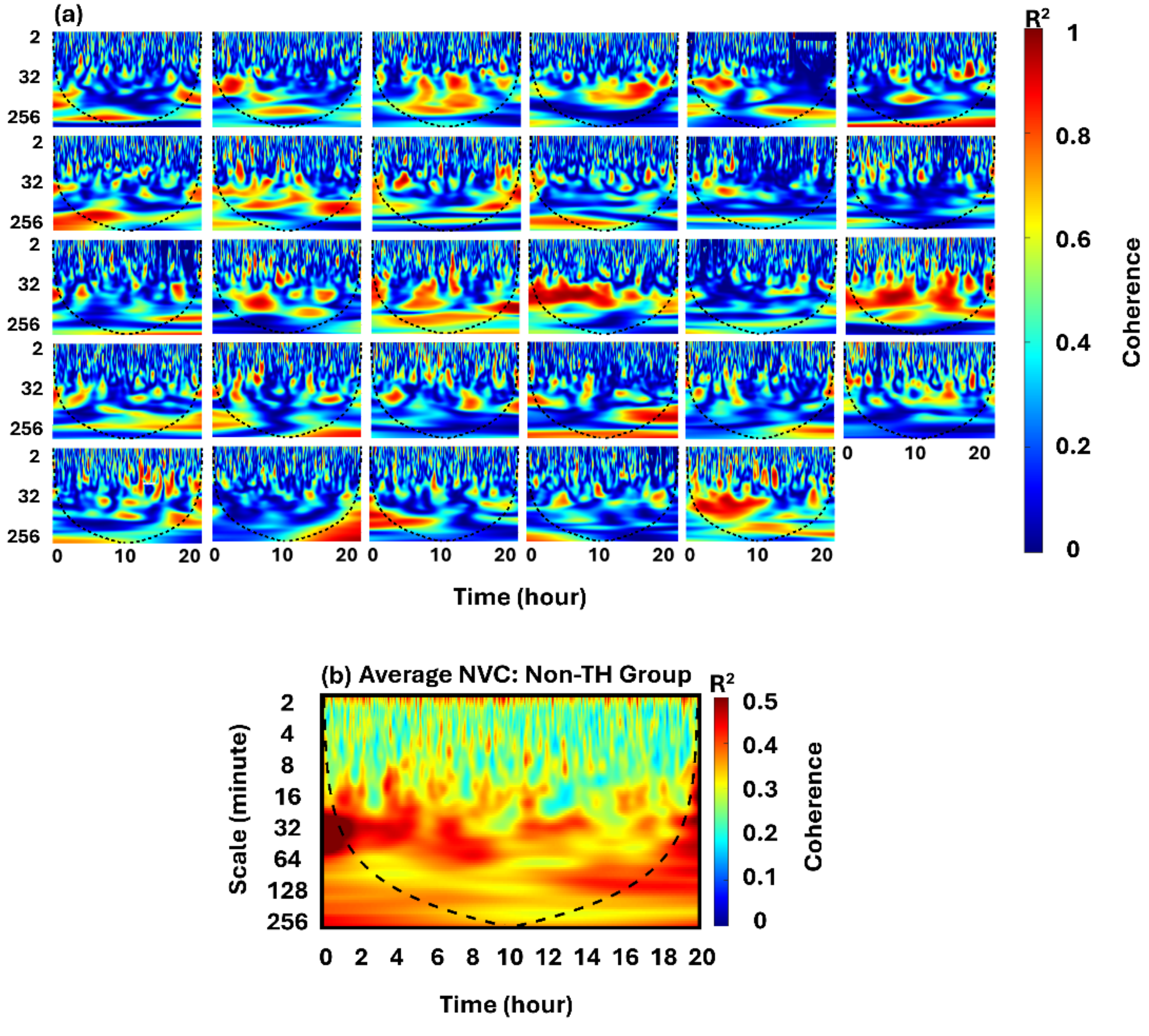

Figure S1. WTC analysis for the non-TH group. (a) Individual WTC maps for all 29 non-TH subjects. (B) Group-averaged coherence map obtained by averaging WTC values across all 29 subjects.

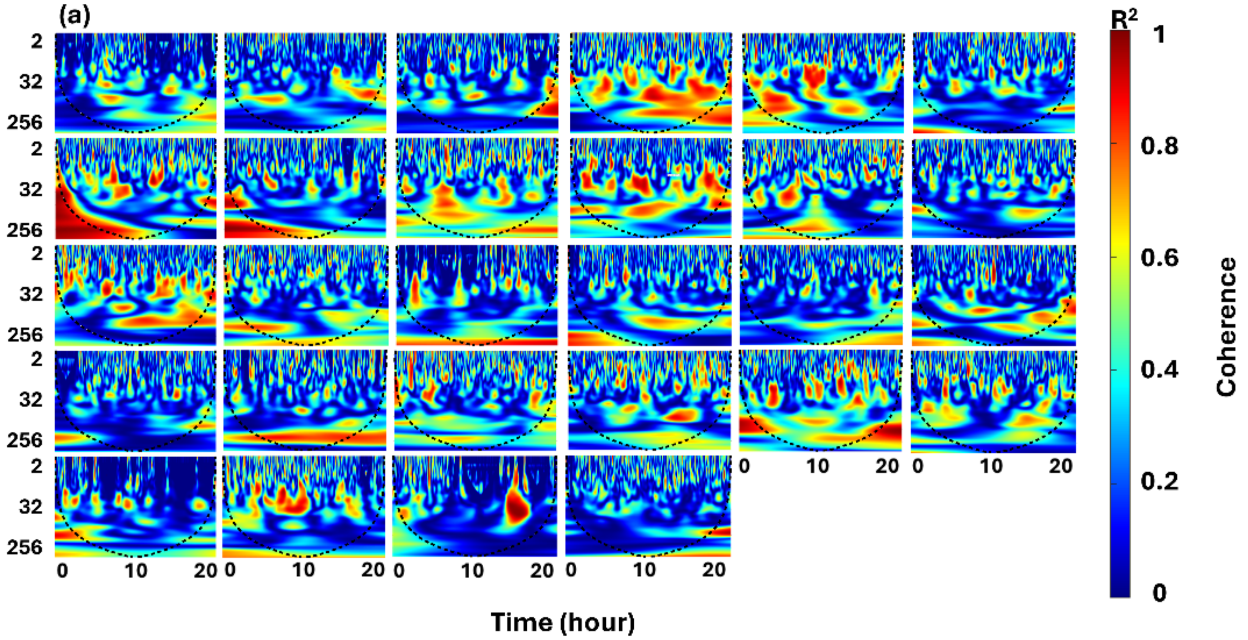

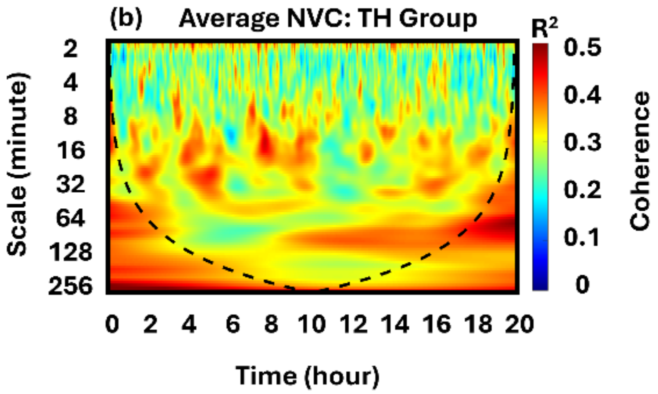

Figure S2. WTC analysis for the TH group. (a) Individual WTC maps for all 28 TH subjects. (B) Group-averaged coherence map obtained by averaging WTC values across all 28 subjects.
